# Research on Modeling and Motion Optimization for an Underactuated Bionic Scorpion Robot Arm

**DOI:** 10.1155/2024/9439878

**Published:** 2024-05-31

**Authors:** Ning Cao, Jinsheng Liu, Feiling Shen, Xuming Pei, Yupu Liu, Hengyu Li

**Affiliations:** ^1^College of Mechanical and Electrical Engineering, Zhengzhou University of Light Industry, Zhengzhou 450002, China; ^2^School of Electrical and Information Engineering, Henan University of Engineering, Zhengzhou 451191, China; ^3^School of Mechatronic Engineering and Automation, Shanghai University, Shanghai 200444, China

## Abstract

Flexible screen possessing a high-contrast ratio to accomplish high-definition display can be attributed to the accuracy of polarizer assembly. Different from the existing polarizer attached onto a flexible screen, resulting in low-accuracy and nonalignment attaching behaviors, a unique approach of underactuated bionic scorpion robot arms can be developed to explore the stable capture and accurate alignment motion operations. Overall structure design and D–H kinematic simulation of bionic scorpion robot arm can be conducted to analyze virtual three-key typical motions. Motion optimization of structural parameters and corresponding motion workspaces verification can be adopted to evaluate motion range and ability. Experiments can be utilized to verify the rationality of theoretical modeling and optimization simulation of bionic scorpion robot arm. Experimental results illustrate that it is evident to demonstrate that the motion behaviors of bionic scorpion robot arm can be verified to be consistent with three key states of virtual theoretical motions. The key joints possessing minor errors may also be utilized to illustrate the relatively excellent dynamic motions existing in bionic scorpion robot arm. Further, advancing the stable motion behaviors of bionic scorpion robot arm may solve the problems of low-accuracy and misalignment polarizer attachments.

## 1. Introduction

Flexible screen, regarded as a key device in the flexible display, can be mainly utilized to accomplish free curved shapes, which are widely applied in many potential critical situations, such as flexible liquid crystal display [1], flexible backplanes [2], biomedical monitoring diagnostics [3], flexible organic light-emitting diode (OLED) display [4], wearable device displays [5] and foldable mobile terminals [6], and so on. Flexible displays are mainly achieved by electronic paper and OLED technologies. Among them, an effectual OLED screen can realize flexible [7], ultra-thin [8], ultra-light, and unbreakable behaviors, so it is broadly employed to realize the variable and bendable operations [9]. Flexible display realized by a flexible screen needs to keep the high contrast [10, 11], which can be attributed to the whole black effect by an attached polarizer [12, 13]. Obviously, an effective polarizer attachment becomes more particularly critical in a polarizer assembly. At present, most of the artificial attachment of polarizers used in the market may generate some disadvantages, such as poor alignment accuracy and low patch efficiency. Moreover, it may cause air outside to flow rapidly into the pressure-sensitive adhesive layer during the assembly of the polarizer onto the flexible screen. This will produce a harmful display containing more shining or darkening behaviors [14, 15]. Although some attachment equipments produced by professional companies are used in attaching polarizers onto flexible screen, but manual operation and semiautomatic facility can lead to low production efficiency, dirt control, attachment accuracy, and so on. This may result in high cost and poor assembly effect during several times of attaching and clipping operations. Additionally, existing methods may present some limitations and shortcomings for polarizer attachment onto flexible screens, including low flexible attaching accuracy, poor alignment, adverse attachment reliability, and poor shape adaptation of application. Correspondingly, it is an urgent need to attempt to address the high quality and adaptive motions for a polarizer attached onto flexible screens well.

Due to robotic motions having the advantages of high precision, high resolution, and flexible operation, it can be utilized to perform assembly behaviors [16]. This may promote a positive effect on polarizer attachment to realize more precise positioning operation and higher patching quality. Generally, existing robotic approaches for polarizer attachment can mainly be divided into an automated robot and visually guided robot operations, where the automated robot is usually custom-made designed to fit a screen with a specific shape, so as to result in the limited screen size, low-accuracy, and narrow scope of application [17]. Visually guided robots, with computer vision technique, can position the screen and the polarizer direction, so as to adjust motions to realize the precise attachment [18]. This method can achieve the higher accurate positioning, higher flexibility, and a wide scope of application. Therefore, considering high precision and flexible for attaching polarizers, it is required to design an adaptive robot to satisfy the higher quality attachment motions.

Naturally, scorpions controlled to achieve the accurate capture of prey, possessing a pair of forceps holders, a tail, and slit sensillum, can exhibit the characteristics of high stable attack accuracy [19], high motion acceleration efficiency [20], strong maneuverability [21] and flexible sensing [22]. Especially, forceps holders can express a highly stable motion behavior to cooperate achievement of alignment operations. This may be utilized to enhance the motion performances to explore the robotic stable capture and accurate alignment behaviors. Although most of bionic scorpion robots can be often used in medical surgery with high precision motion, but this may result in a simple action and large rigidity [23, 24]. As a result, underactuated motion with rope and pulley drive mode may possess a high adaptability and flexibility during mechanical contractile motion [25, 26]. Furthermore, the underactuated mechanism can also have the fast grasping motion with the fine shape adaptability [27].

In order to attempt to address the effects of low-accuracy and nonalignment behaviors on the existing polarizer attached onto the flexible screen, an approach of underactuated bionic scorpion robot arms can be proposed to pursue the virtual stable capture and accurate alignment motion operations. Theoretical modeling, motion simulations and optimization, and experimental verification can be individually conducted to verify the forthcoming motion behaviors and the key virtual spatial operations. Furthermore, it is deduced to indicate that it could be solved to achieve high-accuracy and alignment polarizer attachments using bionic scorpion robot arm.

This paper aims to attempt to address the problems of low accuracy and misalignment during the attaching polarizer under manual operations; an underactuated bionic scorpion robot arm can be designed with rope-pulley mode. This proposed bionic robot arm can be modeled and made motion optimization to enhance the movement flexibility and response speed. Meanwhile, the attachment motion accuracy can be improved by adding a limiting device at the joint. For the sake of achieving the objectives of the study, on the premise of satisfying the range of motion and the feasibility of action, the structure sizes of bionic robot arm are optimized, and the flexibility of the bionic scorpion robot arm is reflected by the operability index, so that it can respond faster to reach the target position on the premise of satisfying the attachment accuracy. Moreover, adopting automatic operations can enhance productivity and reduce uncertainty and errors generated by manual attachment of polarizers, thereby improving product quality and stability [28]. The proposed underactuated bionic scorpion robot arm can enhance the capability of automated attachment technology.

## 2. Structure Design of Bionic Scorpion Robot Arm

During the process of attaching a polarizer onto a flexible display, it needs to get pursuits of accuracy and stability. The second pair of appendages of a scorpion, named a pincer, can have a good ability of stable capture of prey, which can also carry out more accurate alignment operations to improve efficiency, and thus, in order to accomplish the rapid, stable pincer motions of a bionic scorpion, physiological motion pattern can be simulated using robotic underactuated mode, to pursue a virtual capture and alignment of polarizer attachment. In particular, spatial motion patterns of robot joints seem to be consistent with that of a scorpion pincer.

**Figure 1:**
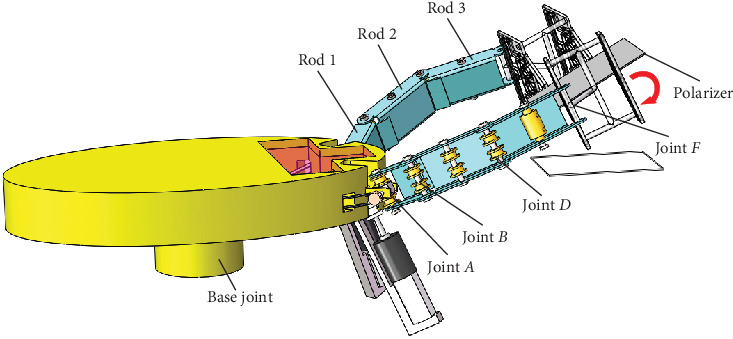
Overall structures of bionic scorpion robot arms with underactuated motion using rope and pulley drive mode.

**Figure 2:**
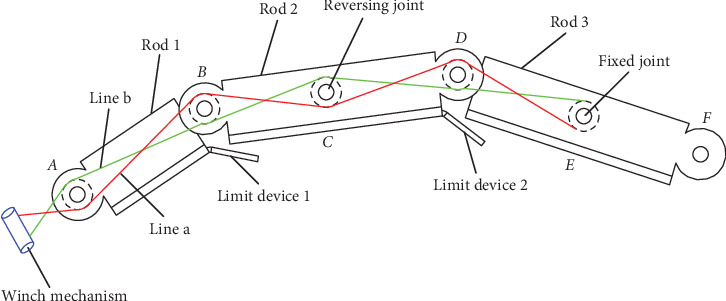
Winding principle of an underactuated bionic scorpion robot arm.

Therefore, overall structures, including the underactuated robot arm, have been developed using rope and pulley drive mode in Figure 1, containing a base joint at the central trunk and two bionic scorpion robot arms.  Figure 2 shows the 8-shaped winding principle of an underactuated bionic scorpion robot arm. Each bionic scorpion robot arm contains three rods, i.e., rod 1, rod 2, and rod 3, where a rotating joint exists between the two rods to complete the rotation behaviors. Each bionic scorpion robot arm contains a rotating joint *A*, a rotating joint *B*, a reversing joint *C*, a rotating joint *D*, a fixed joint *E*, and a rotating joint *F*. Except for rotation motion similar to the other joints, joint *A* can also perform pitching motion, similar to that of the cross universal joint coupling. Although polarizers possess a series of size specifications, they have a certain degree of flexibility. So, a polarizer attached onto a flexible screen may generate a bending situation, more or less. Obviously, it can decrease by a smaller size of polarizers. In order to address a flexible problem, two grippers simultaneously installed at the end of an underactuated bionic scorpion robot arm can be utilized to specially grasp the polarizer. By separately controlling the gripper, it can make more precise rotation adjustments for a virtual alignment. Correspondingly, a polarizer can be adjusted to accomplish an alignment attachment onto a flexible screen. In general, this can achieve a reorientation of the polarizer by the rotation behavior.

Each bionic scorpion robot arm is connected in series by three connecting rods. One end of the rope is fixed on the winch mechanism at the end of the rotating motor and fastened by a plum blossom buckle. The states of rotating joints, including a rotating joint *A*, a rotating joint *B*, a rotating joint *D*, and a rotating joint *F*, can be utilized to quantify the motion behaviors of bionic scorpion robot arm. Rope *a* and rope *b* are interconnected in the form of 8-shaped winding with opposite threading directions. There are two sets of pulleys inside double row structure frames of the robot arm. An extension or bending action of the robot arm can be accomplished by the positive and negative rotation of the motor. An underactuated bionic scorpion robot arm using a rope-pulley combination can realize the self-adaptive motion by two limit devices to realize the linkage motion of each rod. Therefore, because the underactuated bionic scorpion robot arm contains the limit device 1 and limit device 2, it is natural to enhance a high-accuracy motion with a rapid bionic behavior. Additionally, two grippers installed at the end of an underactuated bionic scorpion robot arm can be utilized to make more precise rotation adjustments for a virtual alignment.

After a rope is winded with a closed ring connection, the underactuated bionic scorpion robot arm can be actuated by changing the tension force and position of the rope to accomplish the precise control. The rope-pulley underactuated mode can simplify the transmission link and reduce control difficulty and cost, so the reliability and applicability of the bionic scorpion robot arm can be improved to enhance the motion flexibility. Compared with the full-actuated mode, the rope-pulley underactuated bionic scorpion robot arm can reduce the number of transmission components, friction, and inertia. This may enhance the motion accuracy and stability. Additionally, the attachment accuracy may be improved by the joint limit devices in the underactuated bionic scorpion robot arm.

## 3. Kinematic Modeling and Simulation of Bionic Scorpion Robot Arm

### 3.1. Kinematic Modeling

**Figure 3:**
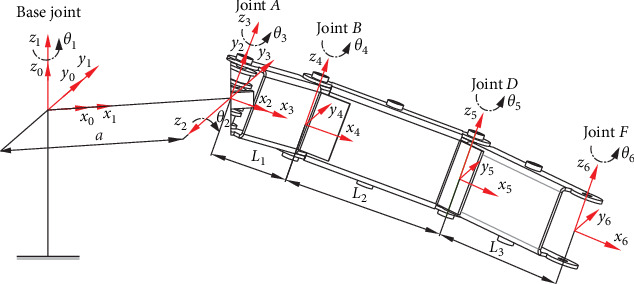
Kinematic coordinate transformation modeling of a bionic scorpion robot arm.

In order to achieve a steady motion for the polarizer attaching onto the flexible screen, structure modeling for optimization of an underactuated bionic scorpion robot arm was conducted by kinematic modeling and analysis. Because of bionic scorpion robot arms having the same two structures and its motion paths, the left of which was taken as an example for kinematic analysis. According to a structural analysis of the bionic scorpion robot arm, the kinematic coordinate transformation is established, as shown in Figure 3. The reference coordinate system constructed by each rotating joint in the robotic arm can be analyzed by the D–H model [29] and preset parameters, which are expressed in Table 1.

Where parameter *i* in Table 1 represents the rod between the coordinate system {*i*−1} and coordinate system {*i*} in Figure 3. In special, parameter *i* = 1, 2, 3 in Table 1 represents the rod between the coordinate system {0} and coordinate system {1}, the coordinate system {1} and coordinate system {2}, the coordinate system {2}, and coordinate system {3}, respectively.

For the sake of more reasonable to conduct theoretical modeling, coordinate transformation relationships of the overall structure can be modeled from the base joint at the central trunk to joint *A* on rod 1. Further, *i* = 6 in Table 1 represents the rod 3 between the coordinate system {5} and coordinate system {6}, but *θ*_6_ represents the rotation of joint *F* in Figure 3. In other words, because joint angle *θ*_*i*_ represents the intersection angle between two common normal lines in an established coordinate system, so *θ*_6_ in Table 1 refers to the rotation angle rotated around axis *z*_6_ from axis *x*_5_ to axis *x*_6_.

Based on the coordinate transformation law of the robotic arm, a homogeneous transformation matrix between two connection rods can be expressed as follows:

(1) $$\begin{array}{c} \begin{array}{c} i^{i-1}T\;=\text{Rot}\left(X,\alpha_{i-1}\right)\text{Trans}\left(X,a_{i-1}\right)\text{Rot}\left(Z,\theta_{i}\right)\text{Trans}\left(Z,d_{i}\right)\\ =\left[\begin{array}{cccc} \cos\theta_{i} & -\sin\theta_{i} & 0 & a_{i-1}\\ \sin\theta_{i}\;\cos\alpha_{i-1} & \cos\theta_{i}\;\cos\alpha_{i-1} & -\sin\alpha_{i-1} & -d_{i}\;\sin\alpha_{i-1}\\ \sin\theta_{i}\;\sin\alpha_{i-1} & \cos\theta_{i}\;\sin\alpha_{i-1} & \cos\alpha_{i-1} & d_{i}\;\cos\alpha_{i-1}\\ 0 & 0 & 0 & 1 \end{array}\right] \end{array}. \end{array}$$

Combined with the rod parameters given in Table 1, the joint space change matrix of the robotic arm can be obtained. Accordingly, the homogeneous the transformation matrix of the robot end relative to the base coordinate system can also be obtained by multiplying the transformation matrix of each rod, i.e., the kinematic equation is as follows:

(2) $$\begin{array}{c} {\text{⁣}}_{6}^{0}T\;={\text{⁣}}_{1}^{0}T\;{\text{⁣}}_{2}^{1}T\;{\text{⁣}}_{3}^{2}T\;{\text{⁣}}_{4}^{3}T\;{\text{⁣}}_{5}^{4}T\;{\text{⁣}}_{6}^{5}T\;=\left[\begin{array}{cccc} n_{x} & o_{x} & a_{x} & p_{x}\\ n_{y} & o_{y} & a_{y} & p_{y}\\ n_{z} & o_{z} & a_{z} & p_{z}\\ 0 & 0 & 0 & 1 \end{array}\right], \end{array}$$

where $[\begin{array}{ccc} n_{x} & n_{y} & n_{z} \end{array}{]}^{T},[\begin{array}{ccc} o_{x} & o_{y} & o_{z} \end{array}{]}^{T},[\begin{array}{ccc} a_{x} & a_{y} & a_{z} \end{array}{]}^{T}$ are the projection components at axis of *x*, *y*, and *z* from robot arm end effector relative to the base coordinate system, respectively. $[\begin{array}{ccc} p_{x} & p_{y} & p_{z} \end{array}{]}^{T}$ can be expressed by translation from the end effector relative to the base.

The elements are represented as follows:

 
*n*_*x*_ = *C*_1_*C*_2_*C*_3456_ − *S*_1_*S*_3456_,
 
*n*_*y*_ = −*C*_1_*C*_2_*S*_3456_ − *S*_1_*C*_3456_,
 
*n*_*z*_ = *S*_2_*C*_3456_,
 
*o*_*x*_ = −*C*_1_*C*_2_*S*_3456_ − *S*_1_*C*_3456_,
 
*o*_*y*_ = −*S*_1_*C*_2_*S*_3456_ + *C*_2_*C*_3456_,
 
*o*_*z*_ = −*S*_2_*S*_3456_,
 
*a*_*x*_ = −*C*_1_*S*_2_,
 
*a*_*y*_ = −*S*_1_*S*_2_,
 
*a*_*z*_ = *C*_2_,
 
*p*_*x*_ = *C*_1_*C*_2_(*L*_3_*C*_345_ + *L*_2_*C*_34_ + *L*_1_*C*_3_) + *a*C_1_ − *L*_3_*S*_1_*S*_345_ − *L*_2_*S*_1_*S*_34_ + *L*_1_*S*_1_*S*_3_,
 
*p*_*y*_ = *S*_1_*C*_2_(*L*_3_*C*_345_ + *L*_2_*C*_34_ + *L*_1_*C*_3_) + *a*S_1_ + *L*_3_*C*_1_*S*_345_ + *L*_2_*C*_1_*S*_34_ + *L*_1_*C*_1_*S*_3_,
 
*p*_*z*_ = *S*_2_(*L*_3_*C*_345_ + *L*_2_*C*_34_ + *L*_1_*C*_3_).
  Where *C*_*i*_ = cos*θ*_*i*_, *S*_*j*_ = sin*θ*_*j*_, *C*_*ij*_ = cos(*θ*_*i*_ + *θ*_*j*_), *S*_*ij*_ = sin(*θ*_*i*_ + *θ*_*j*_).

### 3.2. Typical Motion Simulation

**Figure 4:**
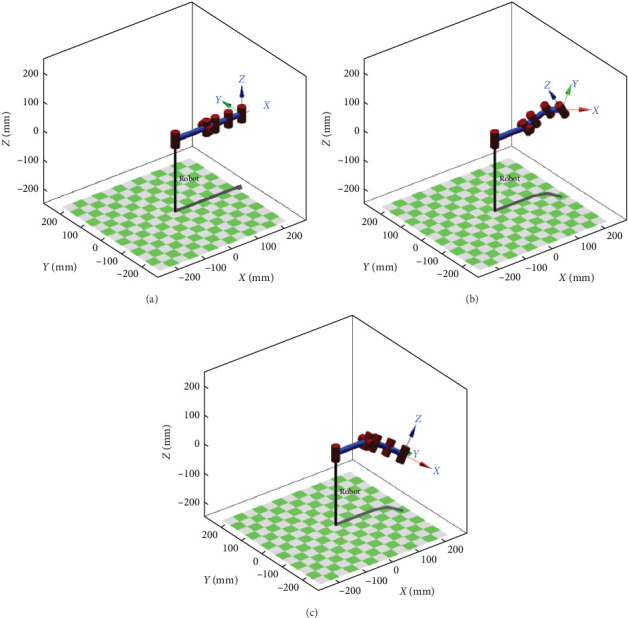
Typical motion simulations of bionic scorpion robot arm: (a) initialized straight state; (b) desired space position and pose for virtual cooperative capture of polarizer; (c) desired space position and pose for virtual cooperative alignment of polarizer.

In order to verify the correctness of kinematic solutions more conveniently and intuitively, robotics toolbox in MATLAB is used for typical motion simulations of bionic scorpion robot arm by the improved D–H parameters [30, 31]. Similarly, it is expedient to take the left arm as an example for motion simulation. Three typical motion simulations of bionic scorpion robot arm are illustrated in Figure 4, including initialized straight state, virtual cooperative capture, and alignment of the polarizer.

When each intersection angle of 0° inputs between bionic scorpion robot arm and the base, the spatial position and pose obtained by MATLAB simulation are consistent with the kinematic calculation result. At this moment, coordinate *T*_0_ = (250, 0, 0) at the initialized straight state in Figure 4(a). Moreover, coordinates of desired space positions and poses for virtual cooperative capture and then alignment of polarizer, as shown in Figures 4(b) and 4(c), are *T*_1_ = (210.925, −54.500, 54.228) and *T*_2_ = (210.925, −54.500, −54.228), respectively. Because the series arm possesses a characteristic of self-adaptation, each joint angle can be fixed after the robot arm has researched the specified position. Therefore, an optimal solution during the position and pose transformation can be found by rod size optimization.

## 4. Size Optimization of Bionic Scorpion Robot Arm

The bionic scorpion robot arm possesses a position adjustment mechanism for capture and alignment of polarizer attachment, and thus, it is obvious that motion performances and structural parameters will affect the dexterity of the robotic arm movement and the adaptability of different operations. Therefore, it is necessary to optimize the sizes of structural parameters to improve the dexterity for smooth operation and fast arrival.

### 4.1. Objective Function Construction

An indication of dexterity for evaluating motion performances can be constructed by singular values of a robot Jacobian matrix, which is utilized to optimize the number of structural steps of the robot. A robot Jacobian matrix describes a linear space relationship of the robot mapped from the joint space velocity to the operation space velocity, which can be expressed as follows:

(3) $$\begin{array}{c} \left[\begin{array}{c} \dot{x}\\ \dot{y}\\ \dot{z}\\ w_{x}\\ w_{y}\\ w_{z} \end{array}\right]=J\left[\begin{array}{c} {\dot{q}}_{1}\\ {\dot{q}}_{2}\\ \vdots \\ {\dot{q}}_{n} \end{array}\right]. \end{array}$$

For a robot with *n* joints, its Jacobian matrix *J* (*q*) is a matrix of order 6 × *n*, where the first three rows can transmit the end linear velocity, and the last three rows are related to the end angular velocity. A Jacobian matrix of bionic scorpion robot arm can be expressed in the form of block matrixes as follows:

(4) $$\begin{array}{c} \left[\begin{array}{c} v\\ w \end{array}\right]=\left[\begin{array}{cccc} J_{l1} & J_{l2} & \cdots & J_{\text{ln}}\\ J_{a1} & J_{a2} & \cdots & J_{an} \end{array}\right]\left[\begin{array}{c} {\dot{q}}_{1}\\ {\dot{q}}_{2}\\ \vdots \\ {\dot{q}}_{n} \end{array}\right], \end{array}$$

where *J*_*li*_ and *J*_*ai*_ represent differential movement and rotation of the end caused by the independent differential movement of joint *i*, respectively.

A Jacobian matrix of the robot can be construed by vector product according to the motion coordinate system. So, a linear velocity *ν* and angular velocity *w* of robot end are related to joint velocity $\dot{q}$. As for a rotating joint *i*, it can be expressed as follows:

(5) $$\begin{array}{c} \left[\begin{array}{c} v\\ w \end{array}\right]=\left[\begin{array}{c} z_{i-1}\times^{0}p_{n}^{i-1}\\ z_{i-1} \end{array}\right]{\dot{q}}_{i},J_{i}=\left[\begin{array}{c} z_{i-1}\times^{0}p_{n}^{i-1}\\ z_{i-1} \end{array}\right], \end{array}$$

where ⁣^0^*p*_*n*_^*i*−1^ = ⁣_*i*−1_^ ⁣⁣  0^*R*^*i*−1^*P*_*n*_^*i*−1^, it can represent a position vector in the base coordinate system {0} of origin of coordinates relative to the coordinate system {*i*−1}. *z*_*i*−1_ is a *Z*-axis single vector in coordinate system {*i*−1}, expressed in the base coordinate system {0}.

In summary, the Jacobian matrix can be obtained as follows:

(6) $$\begin{array}{c} J=\left(\begin{array}{cccccc} J_{1} & J_{2} & J_{3} & J_{4} & J_{5} & J_{6} \end{array}\right), \end{array}$$

where

(7) $$\begin{array}{c} J_{1}=\left[\begin{array}{c} -S_{1}C_{2}\left(L_{3}C_{345}+L_{2}C_{34}+L_{1}C_{3}\right)-aS_{1}-L_{3}C_{1}S_{345}-L_{2}C_{1}S_{34}+L_{1}C_{1}S_{3}\\ C_{1}C_{2}\left(L_{3}C_{345}+L_{2}C_{34}+L_{1}C_{3}\right)+aC_{1}-L_{3}S_{1}S_{345}-L_{2}S_{1}S_{34}-L_{1}S_{1}S_{3}\\ 0\\ 0\\ 0\\ 1 \end{array}\right], \end{array}$$

(8) $$\begin{array}{c} J_{2}=\left[\begin{array}{c} -C_{1}S_{2}\left(L_{3}C_{345}+L_{2}C_{34}+L_{1}C_{3}\right)\\ -S_{1}S_{2}\left(L_{3}C_{345}+L_{2}C_{34}+L_{1}C_{3}\right)\\ C_{2}\left(L_{3}C_{345}+L_{2}C_{34}+L_{1}C_{3}\right)\\ S_{1}\\ -C_{1}\\ 0 \end{array}\right], \end{array}$$

(9) $$\begin{array}{c} J_{3}=\left[\begin{array}{c} -C_{1}C_{2}\left(L_{3}C_{345}+L_{2}C_{34}+L_{1}C_{3}\right)-L_{3}S_{1}S_{345}-L_{2}S_{1}C_{34}+L_{1}S_{1}C_{3}\\ -S_{1}C_{2}\left(L_{3}C_{345}+L_{2}C_{34}+L_{1}C_{3}\right)+L_{3}C_{1}S_{345}+L_{2}C_{1}C_{34}+L_{1}C_{1}C_{3}\\ -S_{2}\left(L_{3}C_{345}+L_{2}C_{34}+L_{1}C_{3}\right)\\ -S_{1}C_{3}-C_{1}C_{2}S_{3}\\ C_{1}C_{3}-S_{1}C_{2}S_{3}\\ -S_{2}S_{3} \end{array}\right], \end{array}$$

(10) $$\begin{array}{c} J_{4}=\left[\begin{array}{c} -C_{1}C_{2}\left(L_{3}S_{345}+L_{2}S_{34}\right)-L_{3}S_{1}C_{345}-L_{2}S_{1}C_{34}\\ -S_{1}C_{2}\left(L_{3}C_{345}+L_{2}C_{34}\right)+L_{3}C_{1}S_{345}+L_{2}C_{1}C_{34}\\ -S_{2}\left(L_{3}S_{345}+L_{2}S_{34}\right)\\ -C_{1}S_{2}\\ -S_{1}S_{2}\\ C_{2} \end{array}\right], \end{array}$$

(11) $$\begin{array}{c} J_{5}=\left[\begin{array}{c} -C_{1}C_{2}L_{3}S_{345}-L_{3}S_{1}C_{345}\\ -S_{1}C_{2}L_{3}S_{345}+L_{3}C_{1}C_{345}\\ -S_{2}L_{3}S_{345}\\ -C_{1}S_{2}\\ -S_{1}S_{2}\\ C_{2} \end{array}\right], \end{array}$$

(12) $$\begin{array}{c} J_{6}=\left[\begin{array}{c} 0\\ 0\\ 0\\ -C_{1}S_{2}\\ -S_{1}S_{2}\\ C_{2} \end{array}\right]. \end{array}$$

Due to an operability of the mechanism, it can not only comprehensively evaluate the motion ability but also measure its overall flexibility [32]. Therefore, an index called operability, $W=\sqrt{\text{det}\left(JJ^{T}\right)}$ defined by Yoshikawa, can be taken as the optimization objective function of the mechanism, which can directly reflect the degrees of the mechanism away from the singular position (*W* = 0) and the indefinite position (*W* ⟶ ∞).

### 4.2. Design Variables and Constraint Conditions

In order to achieve a flexible attachment motion operation, optimization design variables of bionic scorpion robot arm include each rod length *L*_1_, *L*_2_, and *L*_3_. Here, it takes joint angles *θ*_3_, *θ*_4_, and *θ*_5_ as presetting controlled variables. Correspondingly, constraint conditions contain three parts, such as position ranges between joint *A* and joint *F*, minimum and maximum motion ranges of the robot arm, and its minimum required arm size. Specially, it is simplified to set rod *L*_2_ = *L*_3_. Limited by the arm motion range, these can be expressed as follows:

(13) $$\begin{array}{c} \left\{\begin{array}{c} -12\leq L_{1}\;\cos\theta_{3}+L_{2}\;\cos\left(\theta_{3}+\theta_{4}\right)+L_{3}\;\cos\left(\theta_{3}+\theta_{4}+\theta_{5}\right)\leq 133\\ -112\leq L_{1}\;\sin\theta_{3}+L_{2}\;\sin\left(\theta_{3}+\theta_{4}\right)+L_{3}\;\sin\left(\theta_{3}+\theta_{4}+\theta_{5}\right)\leq 112\\ 90\leq L_{1}+L_{2}+L_{3}\leq 133\\ 20\leq L_{1}\leq 50\\ 30\leq L_{2}\leq 50\\ 30\leq L_{3}\leq 50\\ L_{2}=L_{3} \end{array}\right.. \end{array}$$

### 4.3. Evaluation of Optimization Parameters

In order to improve the dexterity, an optimization algorithm, Fmincon, provided by sequential quadratic programing algorithm (SQP) in MATLAB, is used to optimize bionic robot arm parameters. But, from the analysis of the Jacobian matrix, it can be seen that the rod parameters and joint variables will affect the arm dexterities. Because of the required self-adaptive motions, rod parameters can be optimized by the common joint motion angles of *θ*_3_*ϵ* (−*π*/4, *π*/4), *θ*_4_*ϵ* (−*π*/4, *π*/4), and *θ*_5_*ϵ* (−*π*/4, *π*/4). However, if taking the limit devices' motions as joint limited situations, joint motion angles can be actually restricted to be preset *θ*_3_*ϵ* (−15°, −10°), *θ*_4_*ϵ* (20°, 25°) as the joint variables based on the robot arm virtual working process. Therefore, rods optimizations of bionic robot arm are listed by the joint variables, as shown in Table 2. Therefore, rod parameters of robot arm can be optimized by different combinations of joint variables, that is, *L*_1_ = 33 mm, *L*_2_ = 50 mm, and *L*_3_ = 50 mm.

**Figure 5:**
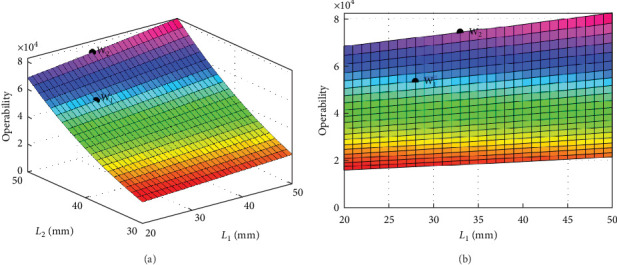
Distributions of the bionic robot arm operability evaluation indexes: (a) combined effects of operability evaluation index at rod *L*_1_ and *L*_2_; (b) projection of operability evaluation index at rod *L*_1_.

Based on the optimized rod parameters, distributions of the arm operability evaluation indexes at different rod lengths are shown in Figure 5. Under the current position and pose, the bionic robot arm does not have a singular position (*W* = 0) and indefinite position (*W* ⟶ ∞). If *W* > 0, the bionic arm may have a nonsingular position. By comparing the previous rod length with its optimizations, it can be demonstrated that an optimized robot arm can also possess the spatial motion ability. Additionally, before the optimization of arm rods, the operability is *W*_1_ = 5.35 × 10^4^. But, it is *W*_2_ = 7.47 × 10^4^ after optimization to increase by 39.6% of the operability index. Therefore, it can be seen that the optimization objective has been achieved to enhance better operability and dexterity.

## 5. Workspace Verification

**Figure 6:**
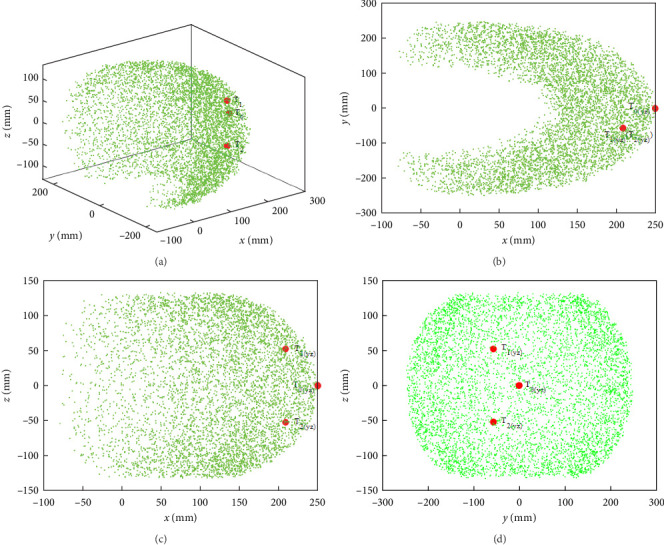
Random motion positions of the left arm in 3D directions: (a) the whole workspace range; (b) a projection in plane *X*–*Y*; (c) a projection in plane *X*–*Z*; (d) a projection in plane *Y*–*Z*.

Due to small, tight, and light underactuated bionic scorpion robot arms possess higher dexterity, its workspace can evaluate motion range and ability. Here, a workspace of the left arm has been described by optimized rod lengths using the Monte Carlo method [33].  Figure 6 shows some random motion positions of the left arm in Cartesian coordinates, including the whole workspace and its projections in 3D directions. Specially, three states of initialized straight *T*_0_, virtual capture of polarizer *T*_1_, and virtual alignment of polarizer *T*_2_ can be selected as the typical working positions of bionic scorpion robot arm, respectively.

In order to achieve the virtual capture and alignment of the polarizer, the real 3D work ranges can be required to design as *x* × *y* × *z* = [210, 250] × [−54.5, 0] × [−54, 55] mm^3^. Actually, it can reach the design requirement in a 3D motion workspace. In a word, the optimized bionic robotic arm can reach the requirements of practical motion ranges and has a high degree of operability.

## 6. Experimental Verification of Bionic Scorpion Robot Arm

### 6.1. Experimental Scheme

**Figure 7:**
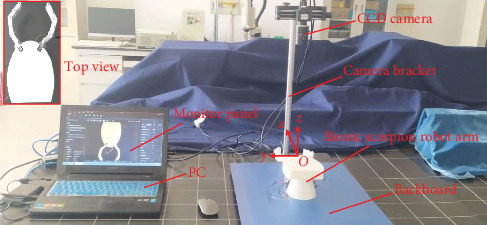
Experimental platform for motion verification of bionic scorpion robot arm. A top view of the bionic scorpion robot arm exhibited on the top left corner.

During the polarizer attachments were conducted by bionic scorpion robot arm, it needed to pursue high-accuracy motions for capture and accurate alignment operations. In order to verify the rationality of theoretical modeling and simulation analysis, experiments can be utilized to analyze the positioning motions and errors.  Figure 7 illustrates an experimental platform for motion verification of bionic scorpion robot arm, and a top view of the bionic scorpion robot arm is exhibited on the top left corner. Taking expedient measurement of joint angles into account, a vision inspection system having image acquisition can be adopted to localize the positions of bionic scorpion robot arm end. Specially, for pursuing the balance between the rapidity and stability of the moving joints in lightweight robot arms, bionic scorpion robot arms were printed for fabrication by a 3D printer. A vision inspection system (MV-CH120-20GC, Hikvision, China) was used for image capture of high-precision plane coordinate recognition and angle detection, and matching parameters were extracted by the LABVIEW platform and vision assistant. During the experiment, by combining the image processing module with the LABVIEW platform, the image features of the key joint motions can be recognized, detected, and located in sequence by running the image signal acquisition and processing programs. Meanwhile, the motion poses were exhibited on the monitor panel in real time, and the motion parameters were displayed accordingly. Thus, experimental joint motion positions data of bionic scorpion robot arm were gathered to compare with simulations when conducting the virtual capture alignment motions.

### 6.2. Experimental Analysis of Bionic Scorpion Robot Arm Key Motion States

During experiments, bionic scorpion robot arm can be first adjusted to realize the initialization operation to become the straight state, and then, according to the relative position relationship for conducting polarizer attachment, it is required to control the bionic scorpion robot arm to accomplish the bending operation to move upward, to achieve the desired space position and pose for virtual cooperative capture of polarizer. While virtual alignment is proceeding, the bionic scorpion robot arm can be controlled to move downward to reach the desired space position and pose for virtual cooperative alignment of the polarizer. Naturally, for the sake of verifying key motion states containing initialized straight state, virtual cooperative capture, and alignment of the polarizer, experimental results can be compared with motion simulations of bionic scorpion robot arm. By detecting and locating the motion features using image processing modules, spatial coordinates at the end of the robot arm were measured to extract joint angles of key motion states. For expediently quantifying bionic robot arm joint angles, each joint at a different state was indicated to analyze the motion behaviors. Specially, the left arm of bionic scorpion robot was designated as an example of quantitative analysis.

**Figure 8:**
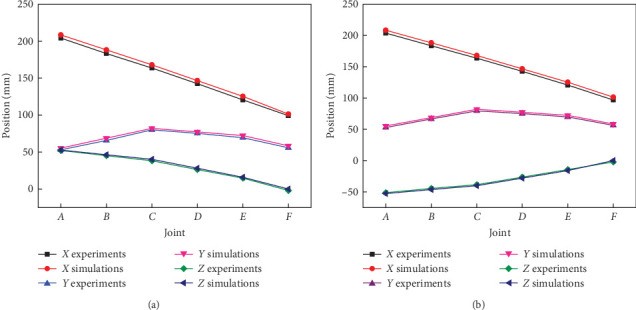
Experimental joint positions comparing with simulations at joints *A*, *B*, *C*, *D*, *E*, and *F* located at the motion state of (a) bionic robot left arm virtual capture and (b) virtual alignment.

Based on visual experimental results of the key motion states, robot arm joint angles can be obtained quantitatively by planar imaging. At the motion state of initialized straight, projection coordinates *F* (213.64, 121.25) were obtained in plane *X*–*Y*. Correspondingly, joint angles at positions *F* and *B* can be individually measured to 178.79° and 182.35°, which seem to be close to 180° under the simulations. In addition, at the motion states of virtual capture and alignment, coordinates of joints *A*, *B*, *C*, *D*, *E*, and *F* were also extracted by two imaging views, respectively, and thus, coordinate values of experimental joint positions at joints *A*, *B*, *C*, *D*, *E*, and *F* can be drawn as compared with the theoretical simulations, as illustrated in Figure 8. It is worth mentioning that *x*, *y*, and *z* indicate the relative spatial location value along 3D directions of robot arm base, respectively. Viewed from Figure 8(a), it was obvious that *Z*-direction heights become increasing from joint *F* to joint *A*, which illustrated that bionic scorpion robot arm possessed the trend to upward behavior at the virtual capture state. On the contrary, viewed from Figure 8(b), bionic scorpion robot arm possessed the trend to downward behavior at virtual alignment. Obviously, it also exhibited a relatively stable motion characteristic from virtual capture to alignment. By quantitative experiments, it is evident to demonstrate that the motion behaviors of bionic scorpion robot arm can be verified to be consistent with three key states of virtual theoretical motions.

Additionally, for the sake of quantifying the key joint angles at joint *B* and joint *D*, spatial vector quantity can be analyzed by space coordinates. That is, if $\vec{a}=\left(x_{1},y_{1},z_{1}\right),\vec{b}=\left(x_{2},y_{2},z_{2}\right)$, there is,

(14) $$\begin{array}{c} \cos<\vec{a},\vec{b}>=\frac{\vec{a}\cdot \vec{b}}{\left|\vec{a}\right|\left|\vec{b}\right|}=\frac{x_{1}x_{2}+y_{1}y_{2}+z_{1}z_{2}}{\sqrt{x_{1}^{2}+y_{1}^{2}+z_{1}^{2}}\sqrt{x_{2}^{2}+y_{2}^{2}+z_{2}^{2}}}. \end{array}$$

So, key joint angles at joint *B* and joint *D* can be calculated, as shown in Table 3. These were close to the theoretical simulation angles of joint *B* 165° and joint *D* 135°, respectively. Therefore, it can also be used to verify a rationality by experimental analysis of the key joint angles at the virtual motion states.

**Figure 9:**
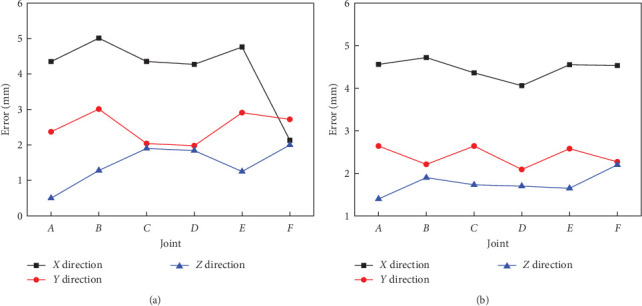
Joint motion position errors between experiments and simulations located at the motion state of (a) left arm virtual capture and (b) virtual alignment.

As a result, joint motion position errors between experiments and simulations can be shown in Figure 9 to illustrate joint deviations at the states of virtual capture and alignment of bionic scorpion robot arm. Viewed from Figure 9(a), joint *F* motion exhibited the largest relative error along the *X* direction, while other joints possessed minor errors. Viewed from Figure 9(b), all of joint motion errors were in a controllable minor range. By contrastive analysis, it is concluded to obtain that the maximum motion position error of bionic robot arm is not more than 3 mm in plane *Y*–*Z*, while it is also not more than 5 mm along the *X* direction. This may be interpreted by the effects of gravity and installation errors on motion dynamic properties at flexible joints. On the whole, the minor motion errors of joint positions can be deduced to verify that bionic scorpion robot arm possesses the relative stable motion behaviors. In addition, different from the relatively poor similarities of passive motion generated by wrist-joint plane motions without regard to the effects of finger extensors and flexors [34], the spatial physiological movement characteristics of bionic scorpion robot arm can be imitated to achieve the relatively complex interactive effects to gain 3D stable and accuracy motions. Furthermore, it can promote a positive effect to quantify the whole motion dynamic properties of bionic scorpion robot arm.

### 6.3. Experiments Analysis of Bionic Scorpion Robot Arm Motion Projection

**Figure 10:**
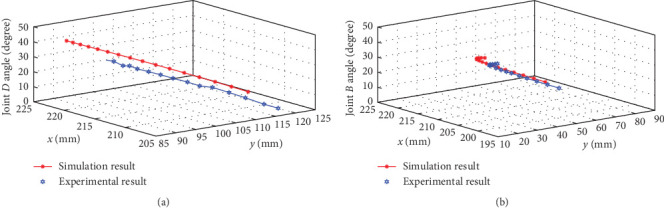
Projection coordinates of motion positions between experiments and simulations of (a) bionic scorpion robot arm joint *D* and (b) joint *B* at different angles.

In order to enhance the motion accuracy, limit device 1 on rod 1 and limit device 2 on rod 2 can be adopted to realize the rapid motions coupled with the accurate motions. The self-adaptive motion can be realized by two limit devices in an underactuated bionic scorpion robot arm using a rope-pulley combination to achieve the linkage motion of each rod. So, the initial operation presents the straight state, and then the bionic scorpion robot arm is controlled to conduct virtual capture motion and alignment motion by means of rope-pulley actuations. Accompanying with operations occurring, joint *D* begins rotating to make rod 3 gradually approach to the limit device 2, and afterward joint *B* begins rotating to make rod 2 gradually approach to the limit device 1. These operation processes exhibit relatively stable motion characteristics, which can also be guaranteed by the two limit devices during relative joint position measurement using the images from the camera. Due to underactuated flexible joints motions applied in bionic scorpion robot arm, controlling variate methods can be adopted to investigate the position accuracy of key joint motions in plane *X*–*Y*. In order to verify the rationality of the key joint *D* and joint *B*, projection coordinates of motion positions of bionic scorpion robot arm joint *D* and joint *B* can be drawn at different angles based on experiments and simulations, as shown in Figure 10. Experimental results demonstrated that the experimental motion positions of key joints were smaller than that of simulations, which may be mainly attributed to the flexible connections during the bionic robot arm virtual motions. In addition, the relative minor deviations from simulations can demonstrate that bionic scorpion robot arm can perform high accuracy of motions. This may be used to interpret flexible joint movements in series that are reasonably applied in bionic scorpion robot arm.

**Figure 11:**
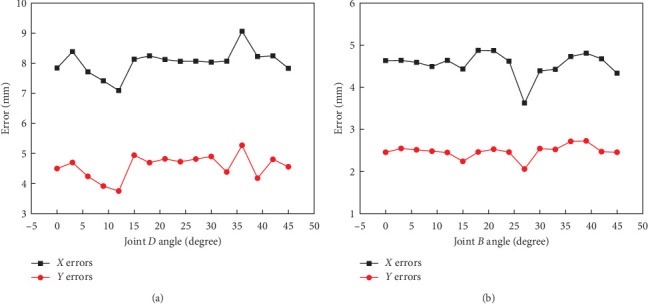
Experimental motion position errors of bionic scorpion robot arm (a) joint *D* and (b) joint *B* at different angles compared with simulations.

In addition, experimental motion position errors of bionic scorpion robot arm joint *D* and joint *B* at different angles can be drawn in Figure 11, as compared with simulations. It should be pointed out that the experimental position errors of robot arm key joints can indicate the relative deviations from motion simulations at different joint angles. Here, an indicator error ratio, as an error metric, represents the percentage value using the relative motion position deviations between the experiments and simulations to compare with the corresponding joint simulation values, and then, the error ratio can be utilized to visually and quantitatively express the relative change of key joint motion error. By analyzing the joint motion errors, it's natural to indicate that joint *D* has an error ratio with maximum 4.08% at the *X*-direction and joint *B* with minimum 2.72% at the *Y*-direction, respectively. These two error ratios at the key joints along different directions can be utilized to analyze the joint sequential motion relations. This seems to be consistent with the state of motion between the joints during the whole operations of bionic scorpion robot arm. Furthermore, according to typical attachment motion requirements, global errors may be restrained to decrease by limit device 1 coupled with limit device 2. Assuming the joint flexibility is not restricted well, the position errors may result in relatively poor motion effects by possible joint flexible deformations after stopping motions without reasonable compensations from structure or movement control [35]. Comparatively speaking, the effects of key joint flexibility may be greatly improved by designing the two limit devices to guarantee the stability for visually grasping manipulations. Correspondingly, the key joints possessing minor error ratios may also be utilized to illustrate the relatively excellent dynamic motions existing in bionic scorpion robot arm. Furthermore, advancing the stable motion behaviors of bionic scorpion robot arm may solve the problems of low-accuracy and misalignment polarizer attachments.

## 7. Discussion

In this paper, an underactuated bionic scorpion robot arm can be classified into a kind of automatic attaching robot. The bionic robot arm adopting rope-pulley actuated mode possesses higher flexibility, and the limit device added at the joint can be utilized to improve the attachment accuracy. This can adapt to a certain range of shapes and sizes of the screen, with a certain degree of versatility and flexibility. Specially, machine vision technology can be further introduced to assist the underactuated bionic scorpion robot arm to complete the attachment process, which can improve the attachment accuracy and efficiency.

Although this method can achieve a smooth motion to guarantee the adhesion behaviors [36], but in fact, it is undeniable that some potential issues or challenges may also be generated assuming the practical implementation of the proposed approach. First, there may be a certain transmission error and friction loss in transmission links, which may result in a slight deviation of the attachment position and angle. Therefore, attachment accuracy may be affected, assuming that the polarizer attaching motions occur. This may be addressed by applying the preload forces to enhance the motion accuracy of the underactuated bionic scorpion robot arm. Second, stable operations of underactuated rope-pulley motions can be achieved by the precision parameter setting and equipment adjustment. However, some manufacturing deviations and deformations affecting motion effects may be limited by the reasonable debugging. Third, once the transmission link is faulty or worn, the robot arm may fail. Therefore, motion security and reliability testing of the underactuated bionic scorpion robot arm may be considered to increase the operational stability. In conclusion, the proposed approach can be utilized to improve the attachment effects. However, it could be continuous optimization and improvement to overcome the practical implementation.

With regard to the criteria and measurement approach to evaluate the accuracy and alignment of the polarizer attachment, it is necessary to be reasonably designed. During the polarizer attachment process, it is required to rotate the polarizer to adjust its polarization direction to align with the linear polarization direction of the upper and lower light sources [37]. The effects of polarizer attachment on the accuracy and alignment mainly include two factors: positional accuracy and angular accuracy. Thus, these are used as the criteria to evaluate the accuracy and alignment of the polarizer attachment by utilizing the underactuated bionic scorpion robot arm, where position accuracy refers to how accurately the polarizer is attached to the target position, and angular accuracy refers to the error between the polarizer's polarization direction and the target angle. Furthermore, the accuracy and alignment of the polarizer attachment can be advanced by real-time stable adjusting the attitude and angle of the underactuated bionic scorpion robot arm. By means of the real-time imaging of the visual device, the motion positions and angles can be extracted to achieve the real-time alignment measurement. Furthermore, motion behaviors may be detected to realize the real-time feedback and attitude adjustment, which can achieve the high accuracy and alignment of the polarizer attachment by utilizing the underactuated bionic scorpion robot arm.

In this paper, the underactuated bionic scorpion robot arm can be utilized to improve the stable and rapid motion properties for the virtual attaching polarizer. However, some limitations may be generated by some relative restriction conditions. First, the weight of the underactuated bionic scorpion robot arm can reduce the effect of the arm's inertia on the positioning accuracy. But it may also produce the vibration and deformation during the attachment motions. Second, the underactuated rope-pulley motion can increase the flexibility of bionic scorpion robot arm. However, this flexibility may result in the nonlinear variation of the arm's trajectory in the motion process to influence the positioning accuracy of bionic scorpion robot arm. Third, the dynamic response ability of bionic scorpion robot arm may be affected by the joint friction, which can decrease the end's positioning accuracy. Additionally, the transmission errors of the arm's joints may be accumulated in the process of motion transmission, which will lead to the lower motion accuracy.

During the polarizer attachment motion process, attachment accuracy and speed can guarantee the quality and yield, respectively. There are some potential areas for future directions where improving the motion range and ability can exhibit more significant areas to optimize the motions of the underactuated bionic scorpion robot arm. For instance, improving the arm's structure to optimize the joint arrangement can change the transmission mode of joint. It can improve the flexibility and stability of the underactuated bionic scorpion robot arm and thus also expand the motion range and ability. Adopting more advanced control algorithms can boost the arm's sensing ability and accuracy to enhance the control ability and adaptability of the underactuated bionic scorpion robot arm. By means of utilizing intelligent methods, the bionic scorpion robot arm can be improved to adapt various scenes to accomplish a polarizer attachment more precisely.

In addition, it may also be explored to expand some other applications for underactuated bionic robot arms. For example, (1) The application of electronic products manufacturing. With the continuous popularization and development of electronic products, the application of robots in the field of electronic products manufacturing become more extensive. The application of this underactuated bionic robot arm in the polarizer attachment has been certain of research and application value. In the future, this bionic robot arm can be used in the manufacturing and assembly process of flexible screens, such as the attachment of touchpads, OLED screens, etc. (2) Biomedical applications. The stable, rapid, and light underactuated bionic robot arm can explore some applications in the surgical robots, assisted rehabilitation equipment, bionic artificial limbs, and other fields. These are utilized to advance some more accurate, safe, and efficient medical services.

## 8. Conclusion

For the sake of trying to figure out the low-accuracy and nonalignment problems in regard to the existing polarizer attachments, a method of underactuated bionic scorpion robot arms can be proposed to pursue the virtual stable capture and alignment behaviors. Overall structure design and D–H kinematic modeling and simulation of bionic scorpion robot arm can be adopted to analyze three key typical motions. It is obvious that an optimal solution during the position and pose transformation can be found by rod size optimization. Then, size optimization of bionic scorpion robot arm structural parameters can be conducted to improve the dexterity for smooth operation and fast arrival, and motion workspaces verification can be utilized to evaluate motion range and ability. It can be seen that optimization objectives have been achieved to enhance better operability and dexterity after increasing by 39.6%. The optimized bionic robotic arm can reach the requirements of practical motion ranges and has a high degree of operability. Experiments can be utilized to verify the rationality of theoretical modeling and simulation analysis of bionic scorpion robot arm. Experimental results illustrate that it is evident to demonstrate that the motion behaviors of bionic scorpion robot arm can be verified to be consistent with three key states of virtual theoretical motions. It can also be used to verify a rationality by experimental analysis of the key joint angles at the virtual motion states. The relative minor deviations from simulations can demonstrate that bionic scorpion robot arm can perform high accuracy of motions. The key joints possessing error ratios, with 4.08% at *X*-directional joint *D* and 2.72% at *Y*-directional joint *B*, may also be utilized to illustrate the relatively excellent dynamic motions existing in bionic scorpion robot arm. Furthermore, advancing the stable motion behaviors of bionic scorpion robot arm may solve the problems of low-accuracy and misalignment polarizer attachments.

## Figures and Tables

**Table 1:** D–H parameters of a bionic scorpion robot arm.

*i*	Torsional angle *α*_*i*−1_ (°)	Rod length *a*_*i*−1_ (mm)	Joint angle *θ*_*i*_ (°)	Joint offset *d*_*i*_ (mm)
1	0	0	*θ*_1_	0
2	90	*a*	*θ*_2_	0
3	−90	0	*θ*_3_	0
4	0	*L*_1_	*θ*_4_	0
5	0	*L*_2_	*θ*_5_	0
6	0	*L*_3_	*θ*_6_	0

**Table 2:** Optimized parameters of bionic robot arm rods.

Number	Presetting joint angle			Optimized rod length		
*j*	*θ*_3_ (°)	*θ*_4_ (°)	*θ*_5_ (°)	*L*_1_ (mm)	*L*_2_ (mm)	*L*_3_ (mm)
1	45	45	45	33.0195	49.9902	49.9902
2	0	45	45	33	50	50
3	0	0	(0, 45]	33	50	50
4	0	[20, 25]	45	33	50	50
5	[−15, −10]	45	45	33	50	50

**Table 3:** Experimental joint angles of bionic scorpion robot left arm.

State	Joint angle	
	Joint *B*	Joint *D*
Virtual capture	163.35°	133.29°
Virtual alignment	165.23°	136.34°

## Data Availability

The data used to support the findings of this study are included within the article.
